# Editorial: Advancing gastrointestinal disease diagnosis with interpretable AI and edge computing for enhanced patient care

**DOI:** 10.3389/fmed.2026.1827042

**Published:** 2026-03-25

**Authors:** Palak Handa, Nidhi Goel, Sri Krishnan, Deepak Gunjan, Anastasios Koulaouzidis, Angel Lanas

**Affiliations:** 1 Research Center for Medical Image Analysis and Artificial Intelligence, Department of Medicine, Faculty of Medicine and Dentistry, Danube Private University, Krems, Austria; 2 Department of Electronics and Communication Engineering, Indira Gandhi Delhi Technical University for Women, New Delhi, India; 3 Department of Electrical, Computer, and Biomedical Engineering, Toronto Metropolitan University, Toronto, ON, Canada; 4 Department of Gastroenterology and HNU, All India Institute of Medical Sciences, New Delhi, India; 5 Department of Clinical Research, University of Southern Denmark, Odense, Denmark; 6 Department of Medicine, Universidad de Zaragoza, IIS Aragón, Zaragoza, Spain

**Keywords:** AI in gastroenterology, clinical decision support systems (CDSS), computing, edge computing, real-time gastrointestinal disease data analysis

For decades, progress in gastrointestinal (GI) diagnostics has followed a familiar pattern: better optics, higher resolution imaging, and increasingly refined classification systems interpreted by trained clinicians (1). Despite remarkable advances in endoscopy and imaging technologies, clinical decision-making has remained fundamentally human-limited (2, 3). Detection depends on attention; interpretation depends on experience; and workflow efficiency depends on time. Modern gastroenterology rarely struggles to capture images; the challenge lies in the clinician's ability to review and interpret the sheer volume produced.

Artificial intelligence (AI) was initially introduced to address this limitation through automated lesion detection. However, the earliest generation of models produced a paradox (3, 4). Although performance frequently surpassed human sensitivity, clinicians were understandably slow to adopt it. Clinicians by and large remain reluctant to delegate decisions to opaque systems whose internal reasoning could not be interrogated, validated at the bedside, or contextualized within clinical judgment. Being correct is not enough; decisions in medicine must be explainable.

The emergence of interpretable (explainable) AI and edge computing represents the point at which medical AI transitions from a research tool to a clinical instrument (Figure 1) (2, 3, 5). Interpretable models expose the reasoning behind predictions, transforming algorithms from silent observers into explainable assistants. Edge computing relocates computation to the device itself, allowing real-time (RT) inference during procedures while preserving privacy and workflow continuity. Together, they redefine the role of AI, allowing it to be seen as an integral partner in the decision-making process. This Research Topic captures that shift across the diagnostic pathway.

**Figure 1:**
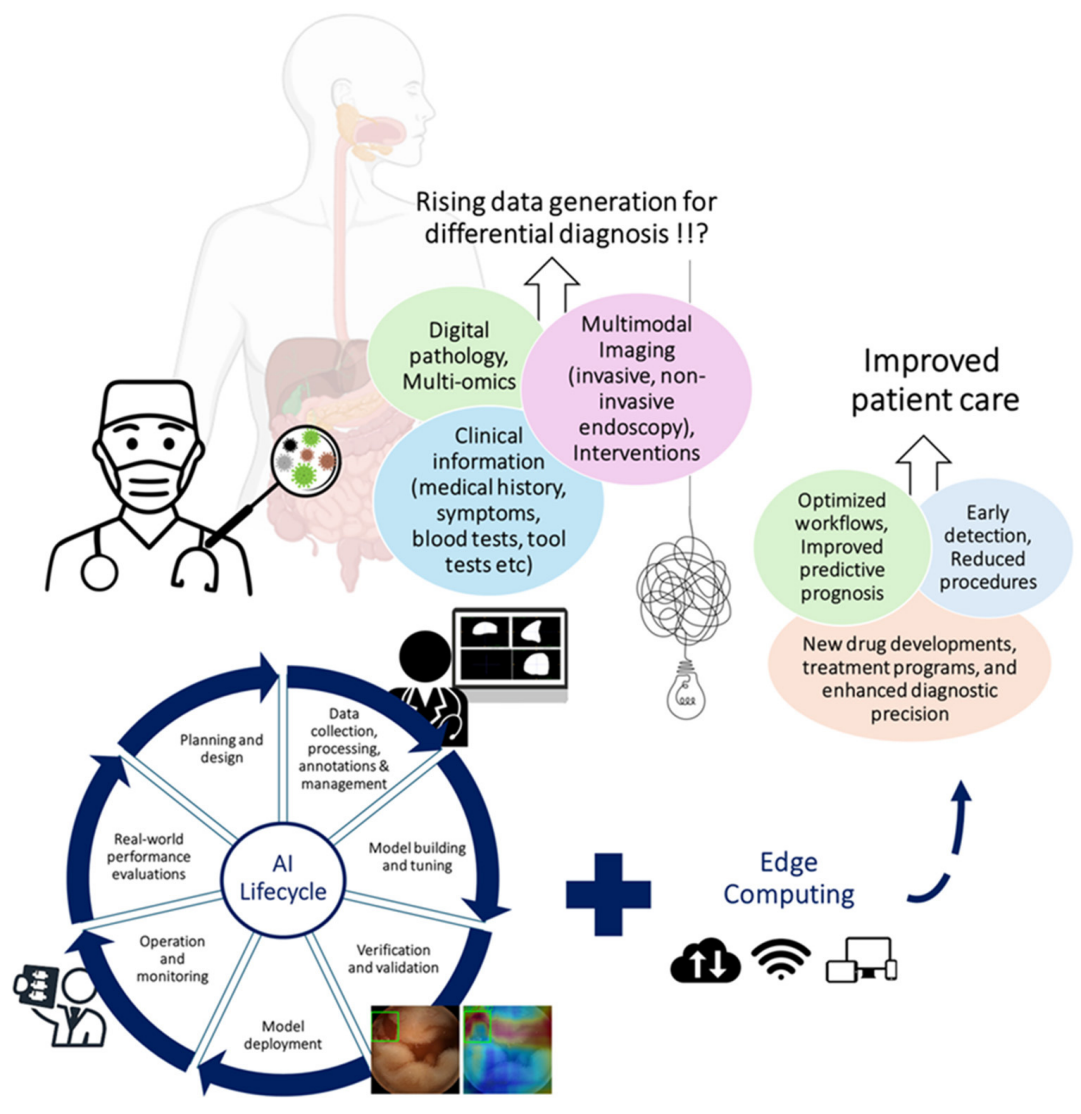
Illustrative diagram highlighting how interpretable artificial intelligence integrated with multimodal data sources and edge computing infrastructures can advance gastrointestinal disease diagnosis, streamline clinical workflows, and ultimately enhance patient care. GI tract element made with BioRender.com.

A first group of studies addresses the acquisition-perception interface. Advanced convolutional networks (ACNs) and segmentation frameworks demonstrate that machine vision can move beyond bounding-box detection toward anatomical understanding, enabling RT identification and delineation of lesions in the esophagus and colon (Pu et al., Yu et al.). Analyses of detection errors reveal that the central problem is under what conditions it fails and how clinicians should interpret those failures (Chang et al.). Parallel developments in image-retrieval (Kim et al.) and optical diagnosis (Bencardino et al.) further illustrate that AI is evolving more toward contextual interpretation, an essential prerequisite for clinical trust.

A second thematic axis concerns prediction rather than perception. Machine learning (ML) models estimating rebleeding risk (Cai et al., Chen et al., Chen and Yang, Duan et al., Zhang et al.), readmission (Wang et al.), and adverse outcomes across diverse gastrointestinal and systemic conditions (Cristea et al.) demonstrate a broader application: AI as a prognostic tool. These approaches shift diagnostics upstream, allowing clinicians to anticipate deterioration rather than react to it. Importantly, the inclusion of interpretable features, transforms predictive scores from statistical abstractions into physiologically meaningful assessments, aligning computational output with medical reasoning.

A third group of contributions bridges physiology, imaging, and systemic disease (Zeng et al., De Cristofaro et al., Hu et al.). Studies exploring metabolic and systemic correlates illustrate the expanding scope of gastroenterology, where intestinal disease is no longer isolated from systemic pathology. Here AI functions not as a replacement for existing clinical scores but as an integrative layer capable of synthesizing heterogeneous variables into coherent risk representations. The goal is not automation of judgment but augmentation of clinical reasoning. Collectively, these studies highlight a fundamental conceptual transition. Early medical AI attempted to replicate the clinician's eye; contemporary AI attempts to support the clinician's decisions. This difference is subtle but decisive. Detection systems improve sensitivity, whereas decision-support systems alter care pathways. The latter requires explainability, because a recommendation must be understood before it can be trusted, and trust precedes adoption in clinical medicine.

Edge computing plays a complementary role. RT inference at the point of care removes the temporal gap between analysis and action. When interpretation occurs during the examination rather than after it, AI becomes part of the procedure itself. The diagnostic act evolves from observation followed by reflection into observation guided by computation. This shift mirrors previous technological transitions in medicine, i.e., from film radiography to digital imaging, and from offline monitoring to continuous telemetry, each redefining workflow rather than merely improving accuracy. The implications extend beyond gastroenterology. Interpretable bedside AI challenges traditional hierarchies of expertise by embedding standardized analytical capability directly into clinical tools. Rather than replacing clinicians, such systems redistribute cognitive effort: machines handle exhaustive pattern recognition, while clinicians concentrate on judgment, context, and patient communication (6). In this framework, AI functions as a cognitive infrastructure rather than a competing decision-maker.

Significant challenges remain. Generalizability across populations, regulatory pathways, and medico-legal accountability will determine the pace of adoption. Progress will also depend on the availability of well-curated open datasets and transparent reporting standards across different modalities. Developing such resources requires shared best practices for data collection and annotation tailored to specific upstream and downstream clinical tasks, with clinicians, scientists, and engineers working together throughout the process. At present, there are no widely accepted global standards for annotating modalities that generate images, signals, text, or videos. As a result, carefully curated expert labels remain the closest thing to a gold standard, since the reliability of any model cannot exceed the reliability of its underlying annotations (7). Even as unsupervised and semi-supervised methods evolve, meaningful and continuous clinical oversight will remain essential.

The next generation of research must therefore move from retrospective performance metrics toward prospective clinical impact, measuring not only diagnostic accuracy but also the practical costs and feasibility of deploying AI in real-time clinical settings where it is most needed, while fostering closer collaboration between clinicians, annotators, and engineers to refine decision-support systems and ultimately improve patient outcomes. Hybrid edge-cloud architectures, multimodal data integration, and continuous learning systems will likely define the coming decade.

This Research Topic illustrates a field approaching clinical maturity. The question is no longer whether AI can interpret gastrointestinal data, but how its reasoning can be aligned with medical practice to generate outputs that are actually useful for clinicians. Interpretable AI provides transparency; edge computing provides immediacy; together, they provide legitimacy. We strongly believe that the future of GI diagnostics will be augmented medicine, i.e., where computation becomes an integral, visible, and accountable participant in clinical care rather than merely a button.

## References

[1] Sinonquel P Vermeire S Maes F Bisschops R. Advanced imaging in gastrointestinal endoscopy: a literature review of the current state of the art. GE-Portug J Gastroenterol. (2023) 30:175–91. doi: 10.1159/000527083 37387720 PMC10305270

[2] Van der Sommen F de Groof J Struyvenberg M van der Putten J Boers T Fockens K . Machine learning in GI endoscopy: practical guidance in how to interpret a novel field. Gut. (2020) 69:2035–45. doi: 10.1136/gutjnl-2019-320466 32393540 PMC7569393

[3] Le Berre C Sandborn WJ Aridhi S Devignes MD Fournier L Smaïl-Tabbone M . Application of artificial intelligence to gastroenterology and hepatology. Gastroenterology. (2020) 158:76–94. doi: 10.1053/j.gastro.2019.08.058 31593701

[4] Urban G Tripathi P Alkayali T Mittal M Jalali F Karnes W . Deep learning localizes and identifies polyps in real time with 96% accuracy in screening colonoscopy. Gastroenterology. (2018) 155:1069–78. doi: 10.1053/j.gastro.2018.06.037 29928897 PMC6174102

[5] Jiang F Jiang Y Zhi H Dong Y Li H Ma S . Artificial intelligence in healthcare: past, present and future. Stroke Vasc Neurol. (2017) 2:230–43. doi: 10.1136/svn-2017-000101 29507784 PMC5829945

[6] Tikhomirov L Semmler C McCradden M Searston R Ghassemi M Oakden-Rayner L. Medical artificial intelligence for clinicians: the lost cognitive perspective. Lancet Digit Health. (2024) 6:e589–94. doi: 10.1016/S2589-7500(24)00095-5 39059890

[7] Nabożny A Balcerzak B Wierzbicki A Morzy M Chlabicz M. Active annotation in evaluating the credibility of Web-based medical information: guidelines for creating training data sets for machine learning. JMIR Med Informat. (2021) 9:e26065. doi: 10.2196/26065 34842547 PMC8665397

